# Mitral Transcatheter Edge-to-Edge Repair in Ventricular Functional Mitral Regurgitation and Very Severe Left Ventricular Dysfunction

**DOI:** 10.1016/j.jacadv.2026.103126

**Published:** 2026-08-14

**Authors:** Kazunori Omote, Masanori Yamamoto, Atsushi Sugiura, Tetsuro Shimura, Shunsuke Kubo, Yuki Izumi, Mike Saji, Masahiko Asami, Yusuke Enta, Shinichi Shirai, Masaki Izumo, Shingo Mizuno, Yusuke Watanabe, Makoto Amaki, Kazuhisa Kodama, Hisao Otsuki, Toru Naganuma, Hiroki Bota, Yohei Ohno, Masahiro Yamawaki, Daisuke Hachinohe, Hiroshi Ueno, Gaku Nakazawa, Toshiaki Otsuka, Kentaro Hayashida, OCEAN-Mitral Investigators

**Affiliations:** a Department of Cardiology, Hokkaido Cardiovascular Hospital, Sapporo Japan; b Department of Medicine II, Kansai Medical University, Osaka, Japan; c Department of Cardiology, Toyohashi Heart Center, Toyohashi, Japan; d Department of Cardiology, Nagoya Heart Center, Nagoya, Japan; e Department of Cardiology, Gifu Heart Center, Gifu, Japan; f Department of Cardiology, Kurashiki Central Hospital, Kurashiki, Japan; g Department of Cardiology, Sakakibara Heart Institute, Tokyo, Japan; h Division of Cardiovascular Medicine, Department of Internal Medicine, Toho University Faculty of Medicine, Tokyo, Japan; i Division of Cardiology, Mitsui Memorial Hospital, Tokyo, Japan; j Department of Cardiology, Sendai Kosei Hospital, Sendai, Japan; k Department of Cardiology, Kokura Memorial Hospital, Kitakyushu, Japan; l Department of Cardiology, St. Marianna University School of Medicine, Kawasaki, Japan; m Department of Cardiology, Shonan Kamakura General Hospital, Kamakura, Japan; n Department of Cardiology, Teikyo University School of Medicine, Tokyo, Japan; o Department of Heart Failure and Transplant Division of Heart Failure, National Cerebral and Cardiovascular Center, Suita, Japan; p Division of Cardiology, Saiseikai Kumamoto Hospital Cardiovascular Center, Kumamoto, Japan; q Department of Cardiology, Tokyo Woman’s Medical University, Tokyo, Japan; r Department of Cardiology, New Tokyo Hospital, Chiba, Japan; s Department of Cardiology, Sapporo Higashi Tokushukai Hospital, Sapporo, Japan; t Department of Cardiology, Tokai University School of Medicine, Kanagawa, Japan; u Department of Cardiology, Yokosuka General Medical Center, Kanagawa, Japan; v Division of Cardiology, Sapporo Cardiovascular Clinic, Sapporo, Japan; w Second Department of Internal Medicine, Toyama University Hospital, Toyama, Japan; x Division of Cardiology, Department of Medicine, Kindai University Faculty of Medicine, Osaka, Japan; y Department of Hygiene and Public Health, Nippon Medical School, Tokyo, Japan; z Department of Cardiology, Keio University School of Medicine, Tokyo, Japan

**Keywords:** left ventricular ejection fraction, mitral regurgitation, mitral transcatheter edge-to-edge repair, outcome, ventricular functional mitral regurgitation

## Abstract

**Background:**

Patients with functional mitral regurgitation (MR) and very severe left ventricular (LV) dysfunction (LV ejection fraction [LVEF] <20%) have been largely excluded from randomized trials of mitral transcatheter edge-to-edge repair (M-TEER), leaving a substantial knowledge gap regarding the role of M-TEER in this high-risk population.

**Objectives:**

This study aimed to evaluate the safety, feasibility, and 1-year outcomes of M-TEER in patients with functional MR and LVEF <20%.

**Methods:**

The OCEAN (Optimized Catheter Valvular Intervention)-Mitral registry prospectively enrolled patients undergoing M-TEER. Among patients with functional MR and LVEF ≤40% (n = 1,538), outcomes were compared between those with LVEF <20% (n = 103) and 20%≤ LVEF ≤40% (n = 1,435). The primary endpoint was the composite of all-cause death and heart failure (HF) hospitalization at 1 year. Secondary endpoints included cardiovascular (CV) death and procedural outcomes.

**Results:**

Acute procedural success was similar between groups (n = 103 [100%, LVEF <20%] vs n = 1,390 [97%, 20%≤ LVEF ≤40%]), with comparable residual MR and postprocedural transmitral pressure gradients. In-hospital mortality did not differ by LVEF category (n = 5 [5%] vs n = 58 [4%]). Both groups showed significant improvement in NYHA functional class at 1 year, although functional status remained worse in patients with LVEF <20%. After adjustment, the primary endpoint did not differ significantly between groups (LVEF <20% vs 20%≤ LVEF ≤40%; HR: 1.24; 95% CI: 0.82-1.88). However, LVEF <20% was independently associated with higher CV mortality (HR: 2.00; 95% CI: 1.08-3.68).

**Conclusions:**

In patients with ventricular functional MR and LVEF <20%, M-TEER was feasible and associated with meaningful symptomatic improvement without excess risk of death or HF hospitalization, although CV mortality remained higher, likely reflecting advanced myocardial disease.

Ventricular functional mitral regurgitation (MR) is primarily a consequence of left ventricular (LV) dilation and geometric remodeling, accompanied by both systolic and diastolic dysfunction, rather than primary valvular pathology.^1^ In this setting, guideline-directed medical therapy (GDMT) represents the cornerstone of management, as optimization of heart failure (HF) therapy may attenuate LV remodeling, improve loading conditions, and reduce regurgitation severity.^2^, ^3^, ^4^ However, in patients with advanced HF and markedly impaired LV ejection fraction (LVEF), GDMT is frequently constrained by hypotension, renal dysfunction, and drug intolerance,^5^^,^^6^ resulting in persistent severe ventricular functional MR despite maximal medical efforts. Consequently, many high-risk patients continue to experience refractory symptoms and recurrent HF hospitalization.^7^

Mitral transcatheter edge-to-edge repair (M-TEER) has emerged as an effective adjunctive strategy to mechanically reduce regurgitant volume and improve clinical outcomes in selected patients with significant ventricular functional MR.^8^^,^^9^ Randomized trials have demonstrated symptomatic and prognostic benefits of M-TEER compared with medical therapy alone,^8^^,^^9^ and subsequent real-world registries have confirmed procedural feasibility and favorable outcomes in broader clinical practice.^10^^,^^11^ These data have established M-TEER as an important therapeutic option for patients with symptomatic ventricular functional MR who remain refractory to optimized medical therapy.

Nevertheless, existing randomized and observational studies have largely excluded patients with very severe LV dysfunction, typically below 20%, leaving a substantial knowledge gap regarding the role of M-TEER in this highest-risk population. Patients with very severe LV dysfunction (LVEF <20%) and advanced LV remodeling have limited therapeutic options beyond medical therapy and advanced HF interventions, and only limited data are available to guide transcatheter treatment decisions. Therefore, the present study aimed to evaluate procedural success, functional improvement, and 1-year clinical outcomes following M-TEER in patients with very severe LV dysfunction (LVEF <20%) compared with those with reduced LVEF (20%-40%), addressing this critical unmet clinical need.

## Methods

### Study population

**Figure 1:**
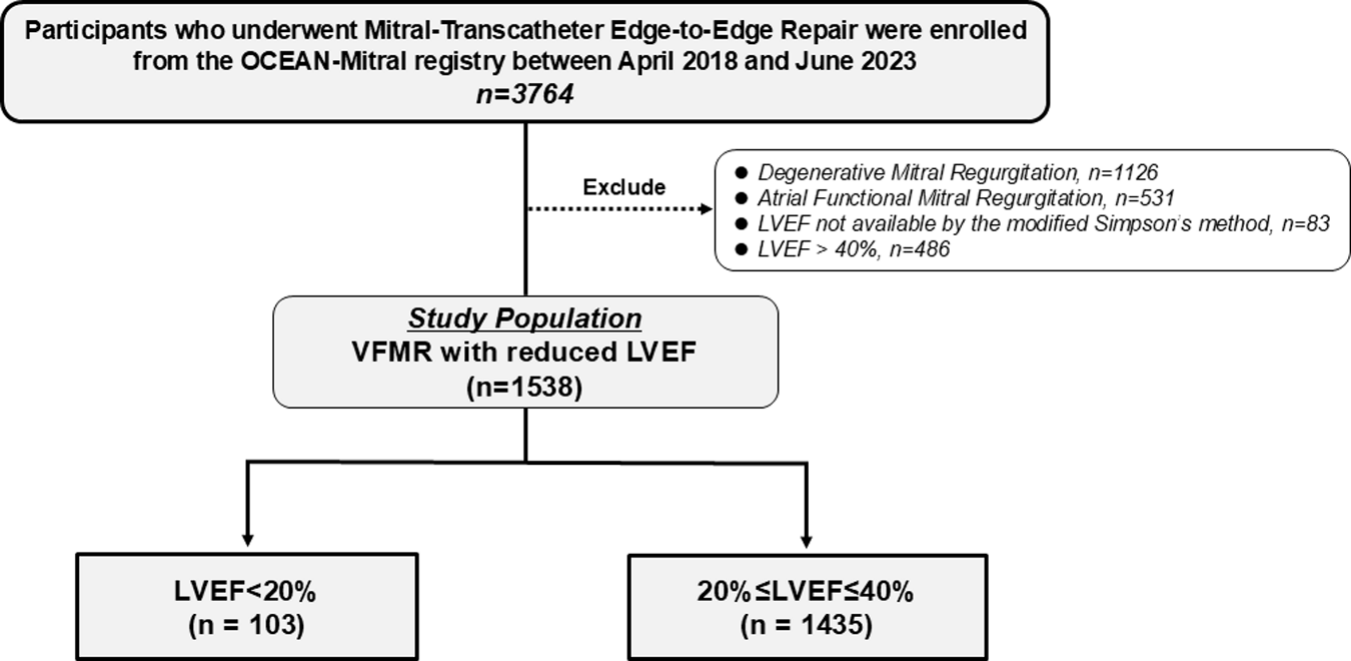
Study Flow Diagram LVEF = left ventricular ejection fraction; OCEAN-Mitral = Optimized Catheter Valvular Intervention Transcatheter Valve Therapies Registry; VFMR = ventricular functional mitral regurgitation.

All data were extracted from the Japanese multicenter OCEAN (Optimized Catheter Valvular Intervention)-Mitral registry.^12^, ^13^, ^14^, ^15^, ^16^, ^17^, ^18^, ^19^ The OCEAN-Mitral registry encompasses 21 Japanese institutions and prospectively enrolled patients who underwent M-TEER for substantial MR. From April 2018 to June 2023, 3,764 patients with significant MR underwent M-TEER as eligible candidates by consensus through discussions among individual heart team members. Patients with degenerative MR (n = 1,126), atrial functional MR (n = 531), LVEF not available by the modified Simpson’s method (n = 83), and LVEF>40% (n = 486) were excluded (Figure 1). Finally, 1538 patients with ventricular functional MR and reduced LVEF (≤40%) were analyzed. The study population was divided into two groups according to LVEF<20% (n = 103) or 20%≤LVEF≤40% (n = 1435). This study was registered with the University Hospital Medical Information Network Clinical Trials Registry via the International Committee of Medical Journal Editors (UMIN000023653). All patients provided informed consent before undergoing M-TEER, and the study protocol was approved by the Institutional Review Board of each participating institution. This study was conducted per the latest version of the tenets of the Declaration of Helsinki and the guidelines for epidemiologic studies issued by the Ministry of Health, Labor, and Welfare of Japan.

### Data collection and definition

Baseline characteristics, laboratory data, and echocardiographic data were prospectively collected using a standardized case report form. The biplane Simpson disk method, from the apical 4- and 2-chamber views, was used to calculate LV end-diastolic volume, LV end-systolic volume, and LVEF. The severity of MR and transmitral pressure gradient (TMPG) was determined based on qualitative and quantitative criteria according to the American Society of Echocardiography (ASE) guidelines. Pulmonary venous flow patterns were assessed by transesophageal echocardiography (TEE) using pulsed-wave Doppler and classified according to the guideline.^20^ The preprocedural and postprocedural MR severity was classified as 0+ (none/trivial), 1+ (mild), 2+ (moderate), 3+ (moderate to severe), and 4+ (severe). The TMPG was measured using continuous Doppler waveform analysis of the mitral diastolic inflow, as outlined in the guidelines.^16^ In patients with atrial fibrillation, the average value was calculated from 3 to 5 consecutive beats.

### Mitral transcatheter edge-to-edge repair procedure

During the study period, the MitraClip device (Abbott Vascular) was the only commercially available mitral transcatheter device in Japan. After introducing the MitraClip Generation (G)2 system, the latest version of the G4 system (without introducing the G3 system) was launched in September 2020. The MitraClip G4 system has 4 different size variations (NT, NTW, XT, and XTW) that aim to adapt to individual patients’ different mitral valve morphologies. M-TEER is indicated in patients with severe MR, regardless of the MR cause. Baseline MR severity was assessed based on the guidelines of the ASE.^20^ Individual heart team members considered the appropriateness of M-TEER based on patient background, including age, frailty, and operative risk of cardiac surgery. The detailed M-TEER procedure has been reported previously.^10^ All M-TEER procedures were performed under general anesthesia under TEE guidance. An acceptable MR reduction after clipping the mitral valve was defined as MR ≤2+ using perioperative TEE findings. If further MR reduction was required, the physicians attempted to change the position of the first clip or implant a second or third clip, depending on the situation. The acute procedural success (APS) of M-TEER was determined as maintenance of procedural safety without life-threatening complications and an adequate reduction of MR ≤2+ in TTE at discharge based on a previous formula and our data. The residual MR was also evaluated by TTE before discharge. All TTE and TEE parameters were calculated based on individual center decision according to the ASE guidelines.^20^

### Clinical outcomes and endpoint

The primary endpoint was 1-year incidence of the composite of all-cause death and HF hospitalization after M-TEER. The secondary endpoints included 1-year incidence of all-cause death, cardiovascular (CV) death, and HF hospitalization, as well as NYHA functional class at 1 year after M-TEER. The CV death and HF hospitalization were assessed using the Mitral Valve Academic Research Consortium criteria.^21^ In-hospital death and procedural adverse events, such as pulmonary complications, access site complication, TEE-associated complications, and acute kidney injury, were compared between patients with LVEF <20% and LVEF ≥20%. APS and the achievement of MR ≤ mild and TMPG <5 mm Hg post M-TEER were also assessed.^16^

### Statistical analysis

Continuous variables are presented as mean ± SD when normally distributed and as medians and IQR when non-normally distributed. Group differences were compared using unpaired *t-*tests or Mann-Whitney *U* tests for continuous variables and chi-square tests or Fisher exact tests for dichotomous variables, when appropriate. Survival curves were estimated by the Kaplan-Meier method and compared with the log-rank test. Cox proportional hazards regression or the Fine and Gray test was used to evaluate the association between LVEF and composite of all-cause death and HF hospitalization, all-cause death, CV death, and HF hospitalization separately, with adjustment for covariates including age, sex, body mass index, coronary artery disease, NYHA functional class, use of inotrope at M-TEER, atrial fibrillation/flutter, estimated glomerular filtration rate, postprocedural TMPG, and post-residual MR. The proportional hazards assumption was confirmed for the Cox proportional hazards models. Missing data were not imputed. Variables with missing values were analyzed using available data only, whereas outcome data were complete for the study endpoints.

A 2-sided *P* value of <0.05 was considered statistically significant. All data were analyzed using JMP14.0 (SAS Institute Inc) and Stata version 15 (StataCorp).

## Results

### Baseline characteristics

Baseline characteristics across LVEF categories are summarized in Table 1. Patients with LVEF <20% were younger, had a lower body mass index, and experienced more HF hospitalizations within 1 year prior to enrollment than in those with LVEF ≥20%. Patients with LVEF <20% had higher EuroSCORE II score, plasma brain natriuretic peptide levels, and required inotropes more frequently. In addition, patients with LVEF <20% exhibited larger LV dimensions and more impaired right ventricular fractional area change. The use of mineralocorticoid receptor antagonists and sodium glucose co-transporter 2 inhibitors was more frequent in patients with LVEF <20% than in those with LVEF ≥20%.

**Table 1:** Patients Characteristics

	Overall (N = 1,538)	LVEF <20% (n = 103)	20%≤ LVEF ≤40% (n = 1,435)	*P* Value
Baseline demographics				
Age, y	74.6 ± 10.0	72.4 ± 8.6	74.8 ± 10.1	0.02
Female	524 (34%)	34 (33%)	490 (34%)	0.81
Body mass index, kg/m^2^	21.4 ± 3.5	20.5 ± 3.1	21.4 ± 3.5	0.01
Coronary artery disease	750 (49%)	53 (51%)	697 (49%)	0.57
Atrial fibrillation/flutter	844 (55%)	56 (54%)	788 (55%)	0.91
Prior HFH within 12 mo prior to enrollment	1,222 (79%)	91 (88%)	1,131 (79%)	0.02
EuroSCORE II, %	6.3 (3.9-10.7)	7.8 (4.4-12.6)	6.2 (3.8-10.6)	0.007
NYHA functional class III or IV	1,059 (69%)	74 (72%)	985 (69%)	0.50
Inotropes at mitral TEER	307 (20%)	31 (30%)	276 (19%)	0.008
eGFR, mL/min per 1.73 m^2^	36 (23-49)	37 (23-55)	35 (23-49)	0.38
BNP, pg/mL	521 (263-1064)	677 (448-1,446)	510 (257-1,034)	0.005
Hemoglobin, mg/mL	11.9 ± 1.9	11.9 ± 1.9	12.0 ± 2.0	0.73
Cardiac structure and function				
Vena contracta, mm	7.0 ± 3.6	7.7 ± 4.9	6.9 ± 3.5	0.09
EROA, cm^2^	0.31 (0.24-0.41)	0.30 (0.22-0.41)	0.31 (0.24-0.41)	0.72
TMPG, mm Hg	1.7 ± 0.9	1.6 ± 1.0	1.7 ± 0.9	0.40
LV ejection fraction, %	29 ± 5	17 ± 3	30 ± 5	N/A
LV end-diastolic dimension, mm	63 ± 9	68 ± 9	63 ± 9	<0.0001
LV end-systolic dimension, mm	55 ± 9	62 ± 10	55 ± 9	<0.0001
LA volume index, mL/m^2^	70 (55-92)	70 (56-93)	70 (55-92)	0.74
TAPSE, mm	15.4 ± 4.5	15.1 ± 4.6	15.4 ± 4.5	0.54
RVFAC, %	33.5 ± 11.3	30.5 ± 13.8	33.7 ± 11.1	0.04
TR pressure gradient, mm Hg	33.6 ± 13.9	33.8 ± 14.6	33.6 ± 13.8	0.90
TR moderate or greater	103 (7%)	7 (7%)	96 (7%)	0.97
Medication				
Renin-angiotensin system blockers	1,034 (68%)	66 (64%)	968 (68%)	0.41
β-Blocker	1,307 (86%)	86 (84%)	1,221 (86%)	0.44
MRA	967 (63%)	75 (73%)	892 (62%)	0.03
SGLT2 inhibitor	470 (31%)	45 (44%)	425 (30%)	0.003
Diuretics	1,311 (86%)	88 (86%)	1,223 (86%)	0.85

Values are mean ± SD, median (IQR), or n (%).

BNP = brain natriuretic peptide; eGFR = estimated glomerular filtration rate; EROA = effective regurgitant orifice area; HFH = heart failure hospitalization; LA = left atrial; LV = left ventricular; LVEF = left ventricular ejection fraction; MR = mitral regurgitation; MRA = mineralocorticoid receptor antagonist; RVFAC = right ventricular fractional area change; SGLT2 = sodium glucose co-transporter 2; TAPSE = tricuspid annular plane systolic excursion; TEER = transcatheter edge-to-edge repair; TR = tricuspid regurgitation; TMPG = transmitral pressure gradient.

### The baseline and improvement of NYHA functional class 1 year after M-TEER

**Figure 2:**
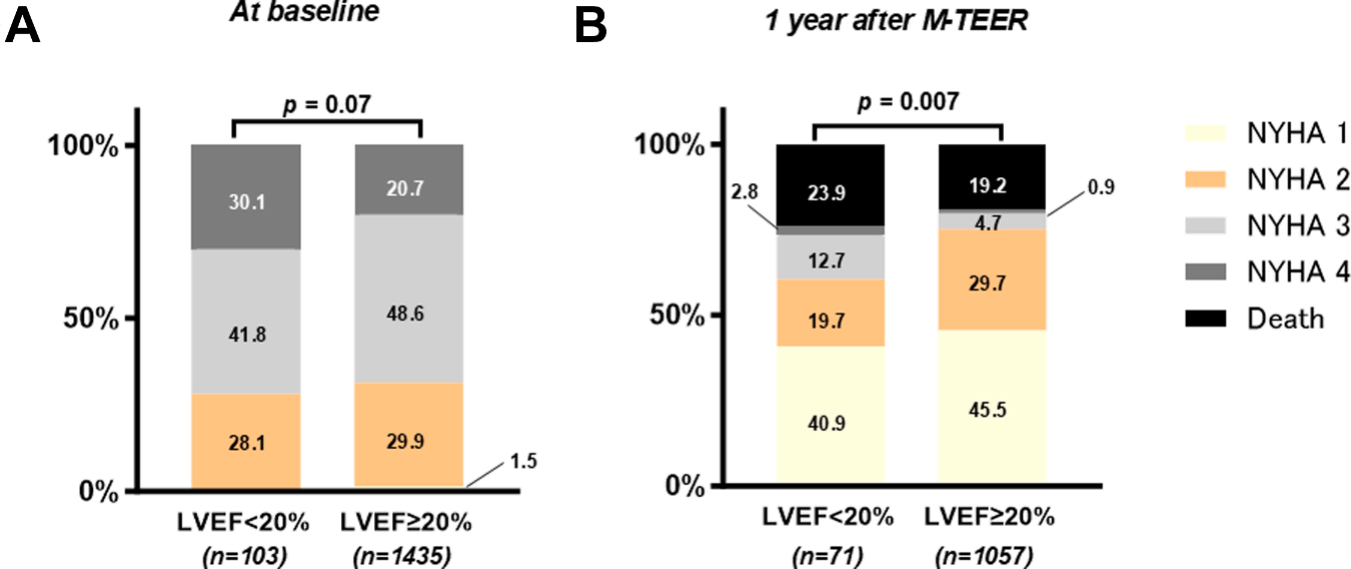
Functional Status at Baseline and 1 Year After Mitral Transcatheter Edge-to-Edge Repair Distribution of NYHA functional class at baseline (A) and 1 year after M-TEER (B) in patients with LVEF <20% and ≥20%. M-TEER = mitral transcatheter edge-to-edge repair; other abbreviation as in Figure 1.

An improvement of NYHA functional class was observed in both the LVEF <20% (*P* < 0.001) and LVEF ≥20% (*P* < 0.001) groups (Supplemental Figure 1). NYHA functional class at baseline was not different between both groups, but that of 1 year after M-TEER in the LVEF <20% group was worse as compared with in 20%≤ LVEF ≤40% group (*P* = 0.007) (Figure 2).

### Procedural results and in-hospital mortality

**Figure 3:**
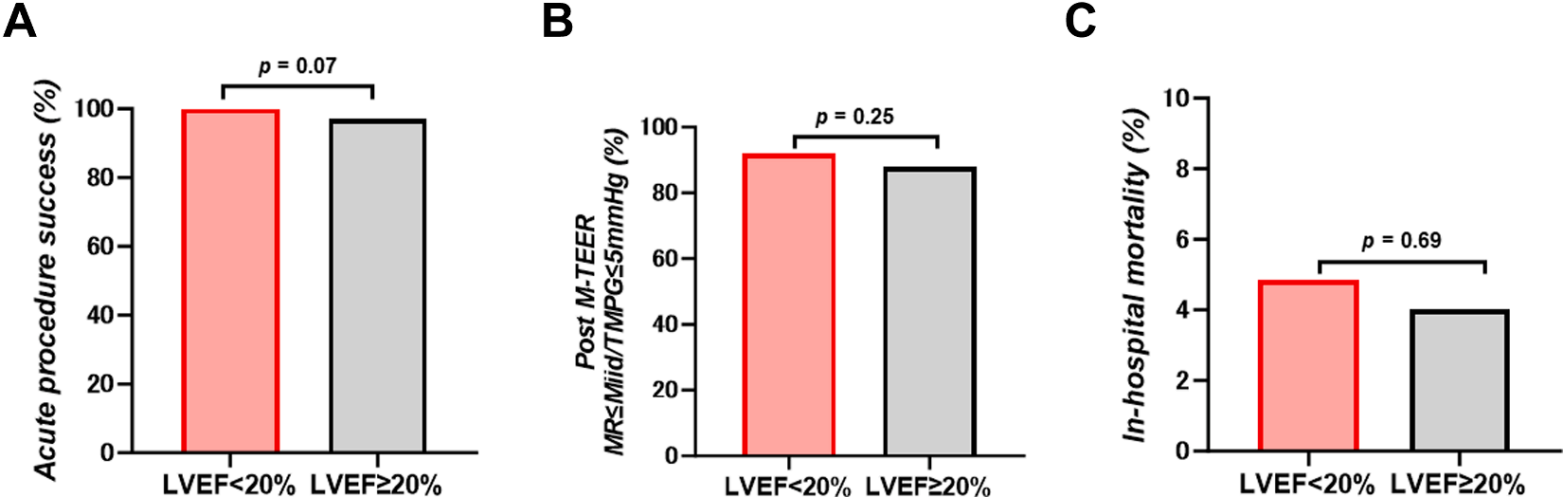
Acute Procedural Success and In-Hospital Mortality by Left Ventricular Ejection Fraction Category The ratio of acute procedure success (A), achieving mild or less mitral regurgitation and postprocedural transmitral pressure gradient <5 mm Hg (B), and in-hospital mortality (C) according to LVEF category. MR = mitral regurgitation; TMPG = transmitral pressure gradient; other abbreviations as in Figures 1 and 2.

Procedural variables are presented in Table 2. Anesthesia times were shorter in patients with LVEF <20% than in patients with 20%≤ LVEF ≤40%. Patients with LVEF<20% slightly fewer clips overall than those with 20%≤ LVEF ≤40%. Regarding clip type, Generation 4 XTW clips were used more frequently as the first implanted device in the LVEF<20% group. APS was achieved in >95% of patients irrespective of LVEF category (Table 2, Supplemental Figure 2). Residual MR grade and postprocedural TMPG were comparable between patients with LVEF<20% and those with 20%≤ LVEF ≤40%. The achievement of mild MR or less/TMPG<5 mm Hg after M-TEER was similar between two groups (Figures 3A and 3B). There was no significant difference in postprocedural pulmonary venous flow pattern between groups. There were no significant differences in procedural-related complications, including major pericardial effusion, single leaflet device attachment, leaflet tear, clip embolization, requirement of iatrogenic atrial septal defect closure, pulmonary, access site–related or TEE-associated complications, and acute kidney injury between the two groups.

**Table 2:** Procedural Results

	Overall (N = 1,538)	LVEF <20% (n = 103)	20%≤ LVEF ≤40% (n = 1,435)	*P* Value
Procedural data				
Anesthesia time, min	140 (108-181)	130 (95-158)	141 (110-184)	0.001
Procedural time, min	78 (57-108)	73 (55-96)	79 (58-110)	0.09
Device time, min	51 (36 to –5)	44 (33-67)	52 (36-75)	0.06
Fluoroscopy time, min	24 (16-35)	28 (18-34)	24 (16-35)	0.39
Acute procedural success, n (%)	1493 (97%)	103 (100%)	1390 (97%)	0.07
Number of clips	1.0 (1.0-2.0)	1.0 (1.0-1.0)	1.0 (1.0-2.0)	0.04
First clip				
Generation 2 NT, n (%)	615 (40%)	34 (33%)	581 (41%)	0.005
Generation 4 NT, n (%)	162 (11%)	6 (6%)	156 (11%)	
Generation 4 NTW, n (%)	415 (27%)	25 (24%)	390 (27%)	
Generation 4 XT, n (%)	26 (2%)	2 (2%)	24 (2%)	
Generation 4 XTW, n (%)	316 (21%)	36 (35%)	280 (20%)	
Second clip				
Generation 2 NT, n (%)	274 (71%)	9 (53%)	265 (72%)	<0.001
Generation 4 NT, n (%)	61 (16%)	0 (0%)	61 (17%)	
Generation 4 NTW, n (%)	22 (6%)	1 (6%)	21 (6%)	
Generation 4 XT, n (%)	16 (4%)	4 (24%)	12 (3%)	
Generation 4 XTW, n (%)	12 (3%)	3 (18%)	9 (2%)	
Third clip				
Generation 2 NT, n (%)	13 (72%)	0 (%)	13 (72%)	N/A
Generation 4 NT, n (%)	3 (17%)	0 (0%)	3 (17%)	
Generation 4 NTW, n (%)	1 (6%)	0 (0%)	1 (6%)	
Generation 4 XT, n (%)	1 (6%)	0 (0%)	1 (6%)	
Generation 4 XTW, n (%)	0 (0%)	0 (0%)	0 (0%)	
Residual MR grade and post-TMPG				
None/trivial, n (%)	465 (30%)	39 (38%)	426 (30%)	0.28
Mild, n (%)	858 (56%)	56 (54%)	802 (56%)	
Moderate, n (%)	170 (11%)	6 (6%)	164 (11%)	
Moderate to severe, n (%)	19 (1%)	1 (1%)	18 (1%)	
Severe, n (%)	22 (1%)	1 (1%)	21 (1%)	
TMPG, mm Hg	2.8 ± 2.1	2.4 ± 1.3	2.8 ± 2.2	0.08
TMPG ≥5 mm Hg, n (%)	123 (9%)	5 (6%)	118 (9%)	0.27
Mild MR or less/TMPG <5 mm Hg	1232 (88%)	82 (92%)	1150 (88%)	0.25
Postprocedural PV pattern				
S-wave > D-wave, n (%)	859 (70%)	64 (76%)	795 (69%)	0.37
S-wave < D-wave, n (%)	344 (28%)	19 (23%)	325 (28%)	
Reverse S-wave	29 (2%)	1 (1%)	28 (2%)	
Procedural-related complications				
Major pericardial effusion, n (%)	7 (0.5%)	0 (0%)	7 (0.5%)	0.48
SLDA, n (%)	15 (1%)	0 (0%)	15 (1%)	0.30
Leaflet tear, n (%)	19 (1%)	0 (0%)	19 (1%)	0.24
Clip embolization, n (%)	0 (0%)	0 (0%)	0 (0%)	N/A
Requirement of IASD closure n (%)	13 (0.9%)	1 (1%)	12 (0.8%)	0.89
Pulmonary complications, n (%)	10 (0.7%)	0 (0%)	10 (0.7%)	0.40
Access site complications, n (%)	23 (1.5%)	0 (0%)	23 (1.6%)	0.20
TEE-associated complications, n (%)	8 (0.5%)	0 (0%)	8 (0.6%)	0.45
Acute kidney injury, n (%)	38 (2%)	3 (3%)	35 (2%)	0.76

Values are mean ± SD, median (IQR), or n (%).

IASD = iatrogenic atrial septal defect; PV = pulmonary venous; SLDA = single leaflet device attachment; TEE = transesophageal echocardiography; other abbreviations as in Table 1.

There was no significant difference in in-hospital mortality between patients with LVEF <20% and those with 20%≤ LVEF ≤40% (Figure 2C).

### One-year outcome after M-TEER

**Figure 4:**
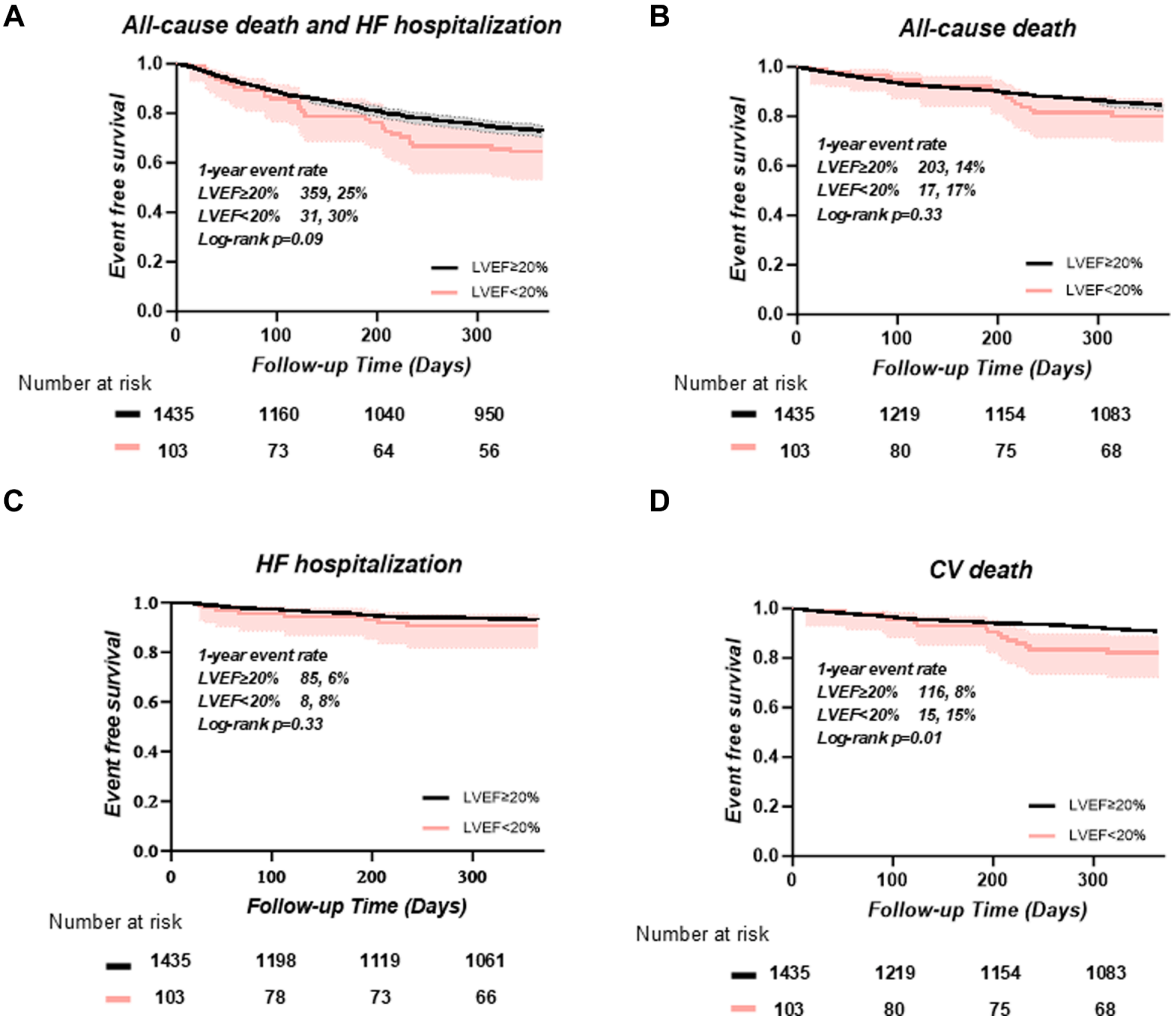
Kaplan-Meier Curves for 1-Year Clinical Outcomes Kaplan-Meier estimates comparing patients with LVEF <20% and LVEF ≥20% for composite of all-cause death and heart failure (HF) hospitalization (A), all-cause death (B), HF hospitalization (C), and cardiovascular (CV) death (D). Abbreviation as in Figure 1.

During the 1-year follow-up period, adverse events occurred in 220 patients (14%) for all-cause death, including 131 CV deaths (9%) and 93 HF hospitalizations (6%). Kaplan-Meier analysis demonstrated no significant difference in event rate for the composite endpoint of all-cause death and HF hospitalization between patients with LVEF<20% (n = 31 [30%]) and those with LVEF ≥20% (n = 359 [25%]) (log-rank *P* = 0.09) (Figure 4A). Similarly, there were no significant differences between the 2 groups (LVEF <20% vs 20%≤ LVEF ≤40%) in all-cause death or HF hospitalization when analyzed separately (n = 17 [17%] vs n = 203 [14%], log-rank *P* = 0.33 and n = 8 [8%] vs n = 85 [6%], log-rank *P* = 0.33, respectively) (Figures 4B and 4C). In contrast, CV death occurred more frequently in patients with LVEF<20% (n = 15 [15%]) compared with those with 20%≤ LVEF ≤40% (n = 116 [8%]) (log-rank *P* = 0.01) (Figure 4D). Similarly, consistent findings were observed at the 2-year follow-up (Supplemental Figure 3). In a sensitivity analysis restricted to patients receiving inotropic support at baseline, results were consistent with the primary analysis, including persistently higher CV mortality in patients with LVEF <20% (Supplemental Figure 4).

In multivariable analyses, LVEF was not independently associated with the composite endpoint of all-cause death and HF hospitalization after adjustment for covariates (per 1% decrease: adjusted HR: 1.01; 95% CI: 0.99-1.03; *P* = 0.18 and LVEF<20% vs 20%≤LVEF≤40%: adjusted HR: 1.24; 95% CI: 0.82-1.88; *P* = 0.32) (Table 3). Similarly, no significant associations were observed between LVEF and either all-cause death (per 1% decrease: adjusted HR: 1.01; 95% CI: 0.99-1.04; *P* = 0.37 and LVEF<20% vs 20%≤LVEF≤40%: HR: 1.28; 95% CI: 0.71-2.28, *P* = 0.41) or HF hospitalization (per 1% decrease: adjusted subdistribution HR[SHR]: 1.03; 95% CI: 0.99-1.08; *P* = 0.16 and LVEF<20% vs 20%≤LVEF≤40%: adjusted SHR: 1.25; 95% CI: 0.42-3.73; *P* = 0.68) when each outcome was analyzed separately.

**Table 3:** Outcomes

	All-Cause Death or HF Hospitalization					
	Univariable			Multivariable		
	Unadjusted HR	95% CI	*P* Value	Adjusted HR	95% CI	*P* Value
LVEF, per 1 decrease	1.02	0.999-1.03	0.06	1.01	0.99-1.03	0.18
LVEF<20% vs 20%≤ LVEF ≤40%	1.37	0.95-1.98	0.09	1.24	0.82-1.88	0.32

	All-cause death					
	Univariable			Multivariable		
	Unadjusted HR	95% CI	*P* Value	Adjusted HR	95% CI	*P* Value
LVEF, per 1 decrease	1.01	0.99-1.03	0.40	1.01	0.99-1.04	0.37
LVEF<20% vs 20%≤ LVEF ≤40%	1.28	0.78-2.09	0.34	1.28	0.71-2.28	0.41

	HF hospitalization					
	Univariable			Multivariable		
	Unadjusted SHR	95% CI	*P* Value	Adjusted SHR	95% CI	*P* Value
LVEF, per 1 decrease	1.02	0.98-1.06	0.27	1.03	0.99-1.08	0.16
LVEF<20% vs 20%≤ LVEF ≤40%	1.35	0.54-3.36	0.52	1.25	0.42-3.73	0.68

	Cardiovascular death					
	Univariable			Multivariable		
	Unadjusted SHR	95% CI	*P* Value	Adjusted SHR	95% CI	*P* Value
LVEF, per 1 decrease	1.05	1.02-1.08	0.002	1.05	1.02-1.08	0.003
LVEF<20% vs 20%≤ LVEF ≤40%	2.02	1.18-3.43	0.01	2.00	1.08-3.68	0.03

Multivariable models were adjusted for age, sex, body mass index, coronary artery disease, NYHA functional class, inotrope at mitral transcatheter edge-to-edge repair (M-TEER), atrial fibrillation/flutter, estimated glomerular filtration rate, post transmitral pressure gradient, and post residual mitral regurgitation. All multivariable models were based on complete-case analysis (n = 1,538).

HF = heart failure; SHR = subdistribution HR; other abbreviation as in Table 1.

In contrast, reduced LVEF was independently associated with a higher risk of CV death. In univariable Fine–Gray analysis, each 1% decrease in LVEF was associated with a 5% increase in the risk of CV death (SHR: 1.05; 95% CI: 1.02-1.08; *P* = 0.002), and patients with LVEF <20% had approximately a two-fold higher risk compared with those with 20%≤ LVEF ≤40% (SHR: 2.02; 95% CI: 1.18-3.43; *P* = 0.01). These associations remained significant after multivariable adjustment (per 1% decrease: adjusted SHR: 1.05; 95% CI: 1.02-1.08; *P* = 0.003; LVEF <20% vs 20%≤ LVEF ≤40%: adjusted SHR: 2.00; 95% CI: 1.08-3.68; *P* = 0.03) (Table 3).

## Discussion

The present study provides novel real-world evidence regarding the role of M-TEER in patients with ventricular functional MR and very severe LV dysfunction (LVEF <20%), a population consistently excluded from randomized trials and incompletely characterized in prior observational studies. There are several key findings in this study. First, M-TEER was performed safely regardless of LVEF severity, with uniformly high APS, comparable MR reduction and postprocedural TMPG, and no significant differences in in-hospital mortality or major procedural complications. Notably, the study period overlapped with the introduction of the MitraClip G4 system. Patients with LVEF <20% received slightly fewer clips overall, whereas Generation 4 XTW clips were more frequently selected as the first implanted device compared with patients with 20%≤ LVEF ≤40%. The increased use of wider G4 clips may reflect evolving device technology and increasing operator confidence in treating patients with severe LV dysfunction. Second, substantial improvement in NYHA functional class was observed at 1 year even among patients with very severe LV dysfunction, demonstrating that advanced LV dysfunction does not preclude symptomatic benefit from M-TEER. However, despite relative improvement, patients with LVEF <20% continued to exhibit worse absolute functional status at 1 year and more severe HF symptoms compared with those with LVEF ≥20%. Third, the composite outcome of all-cause death and HF hospitalization did not differ significantly between LVEF groups, although CV mortality remained higher in the LVEF <20% group. These findings provide important clinical insights into the role of M-TEER in patients with very severe LV dysfunction (LVEF <20%) and inform treatment decision-making in this high-risk population (Central Illustration).

**Central Illustration:**
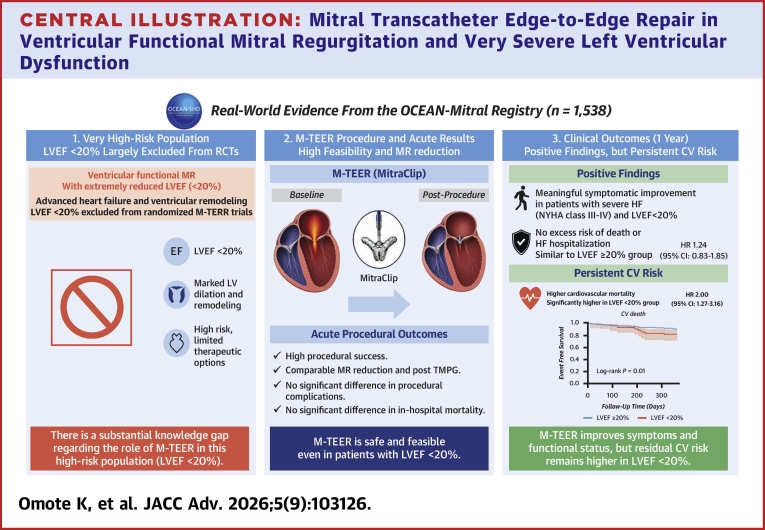
Mitral Transcatheter Edge-to-Edge Repair in Ventricular Functional Mitral Regurgitation and Very Severe Left Ventricular Dysfunction This Central Illustration summarizes the rationale, procedural outcomes, and 1-year clinical outcomes of mitral transcatheter edge-to-edge repair (M-TEER) in patients with ventricular functional mitral regurgitation and very severe left ventricular dysfunction (LVEF <20%) from the OCEAN-Mitral Registry. Despite exclusion from most randomized trials, patients with very severe LV dysfunction achieved high procedural success, effective mitral regurgitation reduction, and significant improvement in functional status after M-TEER. The composite outcome of all-cause death or heart failure hospitalization was comparable between LVEF groups; however, patients with LVEF <20% experienced persistently higher cardiovascular mortality and residual heart failure symptoms, highlighting the need for comprehensive postprocedural heart failure management. CV = cardiovascular; HF = heart failure; RCT = randomized controlled trial; other abbreviations as in Figures 1 and 2.

Existing registry-based outcome data include patients with a broad range of MR etiologies and LV function, making it difficult to delineate outcomes specifically attributable to very severe LV dysfunction.^22^^,^^23^ Among larger registry analyses, the German Transcatheter Mitral Valve Interventions (TRAMI) registry demonstrated high APS and substantial symptomatic improvement in patients with markedly impaired LV function, including those with LVEF below 30%, establishing that M-TEER can achieve meaningful clinical benefit despite advanced myocardial disease.^24^ However, TRAMI included heterogeneous MR etiologies and broad LVEF ranges, limiting definitive conclusions regarding outcomes specifically in patients with very severe LV dysfunction. Similarly, a small observational study in patients with extremely reduced LVEF (<20%, n = 21) has demonstrated high technical success and meaningful functional improvement after M-TEER in selected cases, supporting procedural feasibility even in advanced HF.^25^ Another recent retrospective study specifically evaluating patients with very severe LV dysfunction undergoing M-TEER included 96 patients with a median LVEF of 15%.^26^ Despite the advanced stage of HF, procedural success was achieved in 98% of patients, with sustained MR reduction and a 1-year survival rate of 74%. These findings further support the feasibility of M-TEER in patients with very severe LV dysfunction. However, the absence of a comparator group with less severe LV dysfunction limited assessment of the incremental prognostic impact attributable to very severe LV dysfunction. In contrast, the present study directly compared outcomes between patients with LVEF <20% and those with 20%≤LVEF≤40%, providing further insight into the impact of very severe LV dysfunction on clinical outcomes after M-TEER.

Patients with ventricular functional MR and very severe LV dysfunction often remain highly symptomatic despite maximally tolerated GDMT and are frequently ineligible for advanced HF interventions such as heart transplantation or durable mechanical circulatory support due to age and comorbidities.^27^^,^^28^ The absence of EF-specific evidence has therefore represented a major unmet clinical need. Our findings provide important support for M-TEER as a viable therapeutic strategy to reduce MR burden and improve functional status in this underserved population. The relatively high mortality rates observed in both groups compared with some prior observational studies may reflect the advanced disease stage and high-risk profile of the present cohort. All patients had ventricular functional MR with reduced LVEF and were treated in a real-world setting, including elderly individuals with substantial comorbidity burden. Notably, despite a higher prevalence of inotropic support at the time of M-TEER among patients with LVEF <20% than among those with 20%≤ LVEF ≤40% (30% vs 19%), outcomes in the very severe LV dysfunction group appeared more favorable than might be expected based on prior studies of chronic inotrope-dependent HF. This discrepancy may reflect differences in patient selection, as patients undergoing M-TEER were considered suitable candidates by the local heart team at each participating center.

A decline in LVEF after M-TEER has been reported in a subset of patients following MR reduction, likely reflecting limited myocardial reserve rather than procedural failure. Importantly, previous studies have shown that such postprocedural LVEF decline is not uncommon and is not consistently associated with adverse clinical outcomes.^19^ These results argue against rigid LVEF-based exclusion thresholds and support a more individualized approach to patient selection incorporating ventricular geometry, symptom severity, tolerance to medical therapy, and overall physiological reserve. Future prospective randomized trials specifically enrolling patients with very severe LV dysfunction are warranted to determine whether M-TEER combined with contemporary HF therapy can translate symptomatic improvement into durable prognostic benefit.

### Study Limitations

Several limitations of the present study should be acknowledged. This was an observational analysis derived from a prospective multicenter registry, and therefore residual confounding cannot be fully excluded despite multivariable adjustment. Data regarding advanced HF therapies, including LV assist device implantation and heart transplantation, were not available in the registry. Additionally, echocardiographic assessments were conducted at individual institutions rather than being evaluated by a centralized core laboratory, introducing potential interobserver variability in echocardiographic measurements. Although nearly 80% of patients had experienced HF hospitalization within the year preceding M-TEER, the incidence of HF hospitalization during follow-up was substantially lower. This difference should be interpreted with caution because these measures are not directly comparable and because patients undergoing M-TEER represent a selected population considered suitable candidates by the local heart team. In addition, although CV death was prospectively recorded according to the Mitral Valve Academic Research Consortium criteria, detailed cause-specific classifications were not systematically collected or independently adjudicated. Finally, the present analysis focused exclusively on patients undergoing M-TEER and lacked a contemporaneous medically treated control group, precluding definitive conclusions regarding causal treatment effects. Future prospective randomized trials specifically enrolling patients with very severe LV dysfunction will be required to validate these findings and define the optimal role of M-TEER in this highest-risk population.

### Conclusions

M-TEER achieved high APS and meaningful symptomatic improvement in patients with ventricular functional MR and very severe LV dysfunction. Procedural safety and the composite outcome of all-cause death and HF hospitalization were comparable to those observed in patients with less severe dysfunction, supporting that low EF alone may not preclude consideration of M-TEER. However, persistently higher CV mortality and residual HF symptoms in the very severe LV dysfunction group highlight the advanced disease stage of this population and indicate that M-TEER should be viewed as a complementary strategy for symptom relief rather than a definitive therapy for progressive cardiomyopathy. Prospective randomized studies are warranted to further define long-term clinical benefit and optimal patient selection.

## Funding Support and Author Disclosures

The OCEAN-Mitral registry, which is part of the Optimized Catheter Valvular Intervention–Structural Heart Disease (OCEAN-SHD) registry, was supported by Edwards Lifesciences Japan, Japan; Medtronic Japan, Japan; Boston Scientific Japan, Japan; Abbott Medical Japan, Japan; and the Daiichi Sankyo Company, Japan. Drs Yamamoto, Saji, Izumi, Asami, Enta, Shirai, Izumo, Mizuno, Watanabe, Amaki, Kodama, Bota, Ohno, Hachinohe, Ueno, Kubo, and Hayashida are clinical proctors of TEER for Abbott Medical. Drs Yamamoto, Sugiura, Shimura, Saji, Izumi, Asami, Enta, Shirai, Izumo, Mizuno, Watanabe, Amaki, Kodama, Otsuki, Naganuma, Bota, Ohno, Hachinohe, Ueno, Nakazawa, Kubo, and Hayashida have received lecturer/consultant/advisor fees from Abbott Medical. All other authors have reported that they have no relationships relevant to the contents of this paper to disclose.

Perspectives **COMPETENCY IN PRACTICE-BASED LEARNING:** M-TEER is increasingly used in patients with ventricular functional MR and advanced HF. However, patients with reduced LV ejection fraction (LVEF <20%) have largely been excluded from randomized trials. In the OCEAN-Mitral registry, M-TEER in patients with LVEF <20% was associated with high procedural success and meaningful symptomatic improvement without excess risk of death or HF hospitalization, supporting consideration of M-TEER even in patients with advanced ventricular dysfunction.

**TRANSLATIONAL OUTLOOK:** Although M-TEER improved symptoms in patients with LVEF <20%, CV mortality remained high, likely reflecting advanced myocardial disease. Future prospective randomized studies are needed to determine whether M-TEER combined with contemporary HF therapy can improve long-term prognosis and to refine patient selection beyond conventional LVEF thresholds.
