# Supplementary material for: Mitral Transcatheter Edge-to-Edge Repair in Ventricular Functional Mitral Regurgitation and Very Severe Left Ventricular Dysfunction
Source: JACC Adv. 2026 Aug 14;5(9):103126. doi: 10.1016/j.jacadv.2026.103126 (PMC13482209; doi:10.1016/j.jacadv.2026.103126)
Supplement: Supplementary file 1 [file mmc1.pdf]

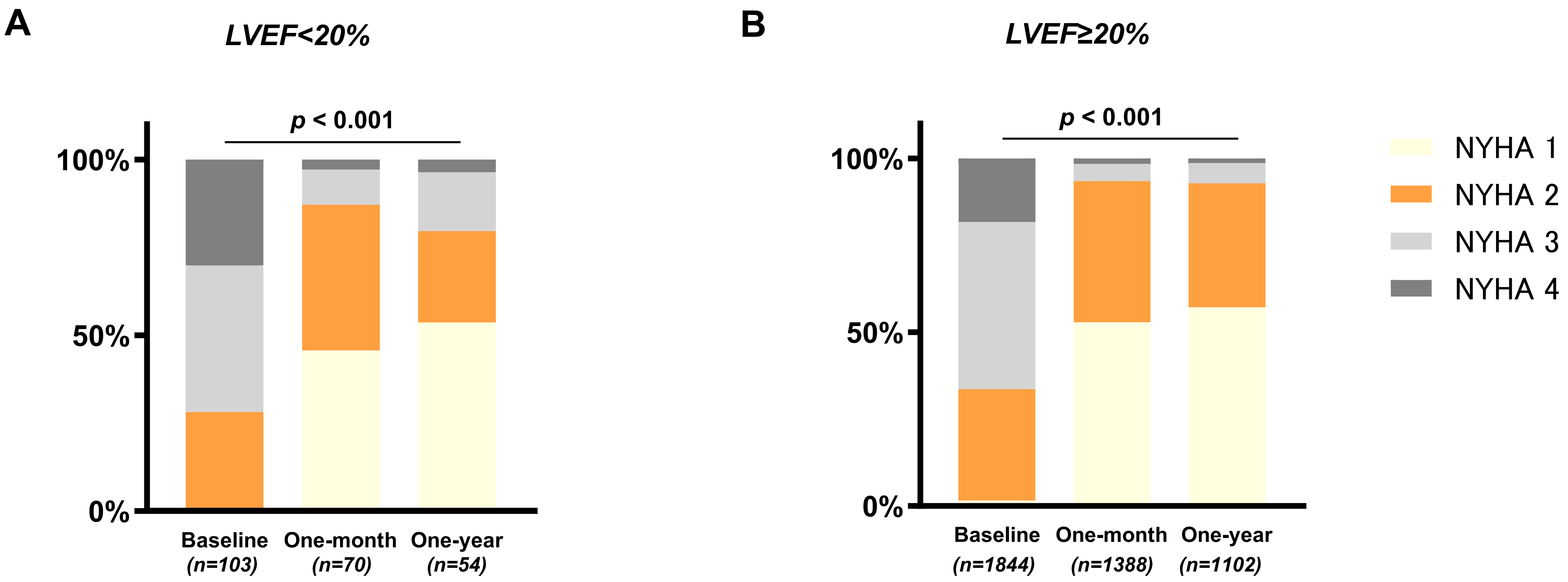

Supplemental Figure 1

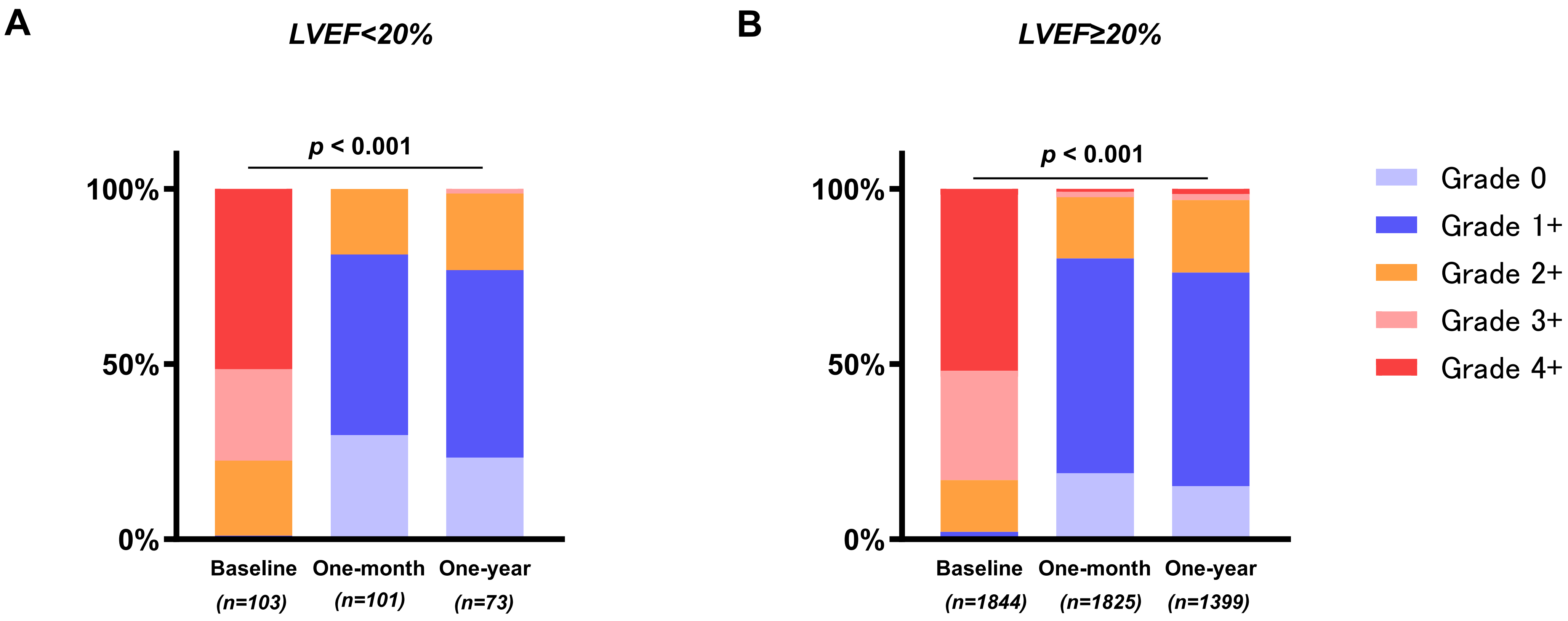

Supplemental Figure 2

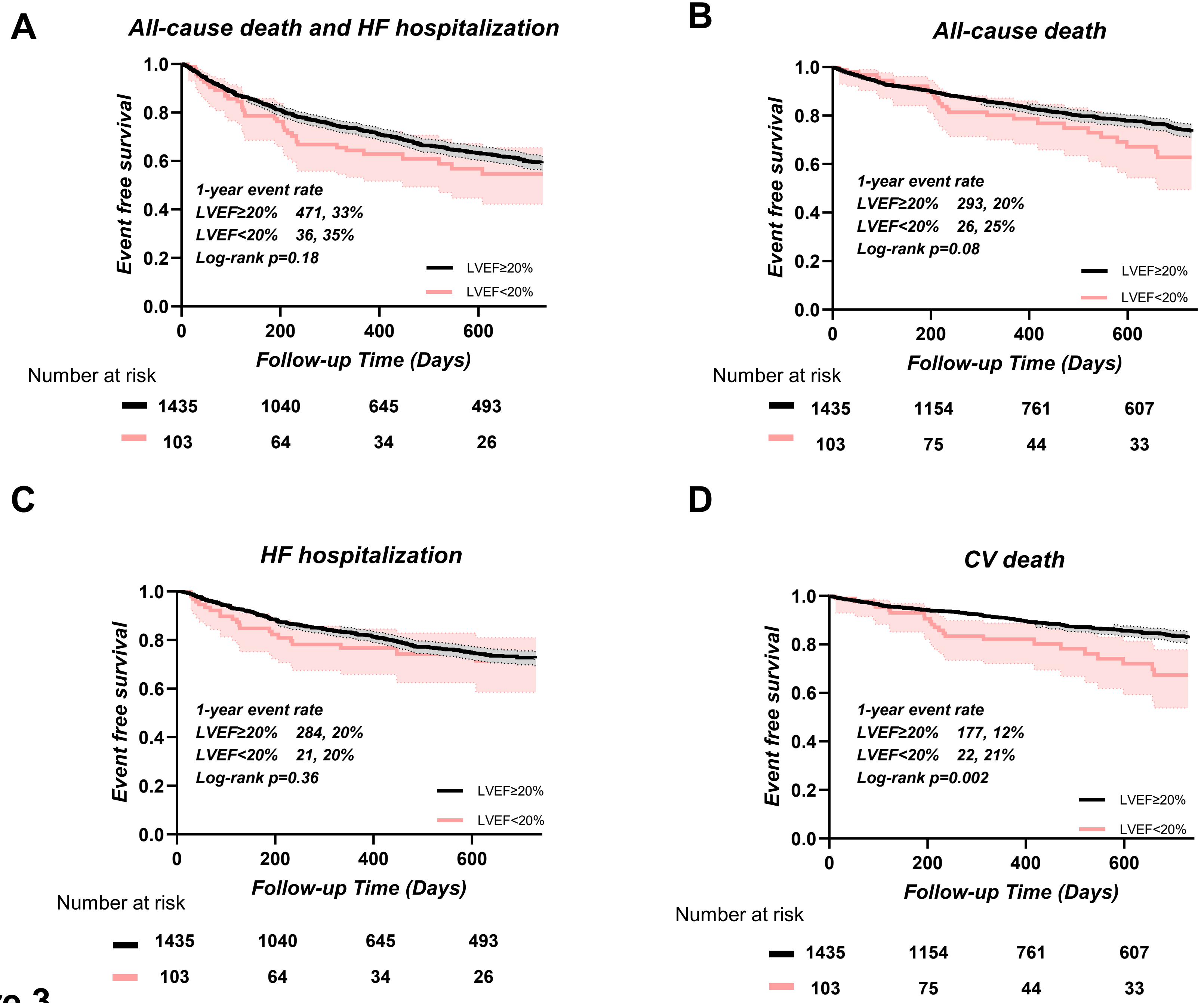

Supplemental Figure 3

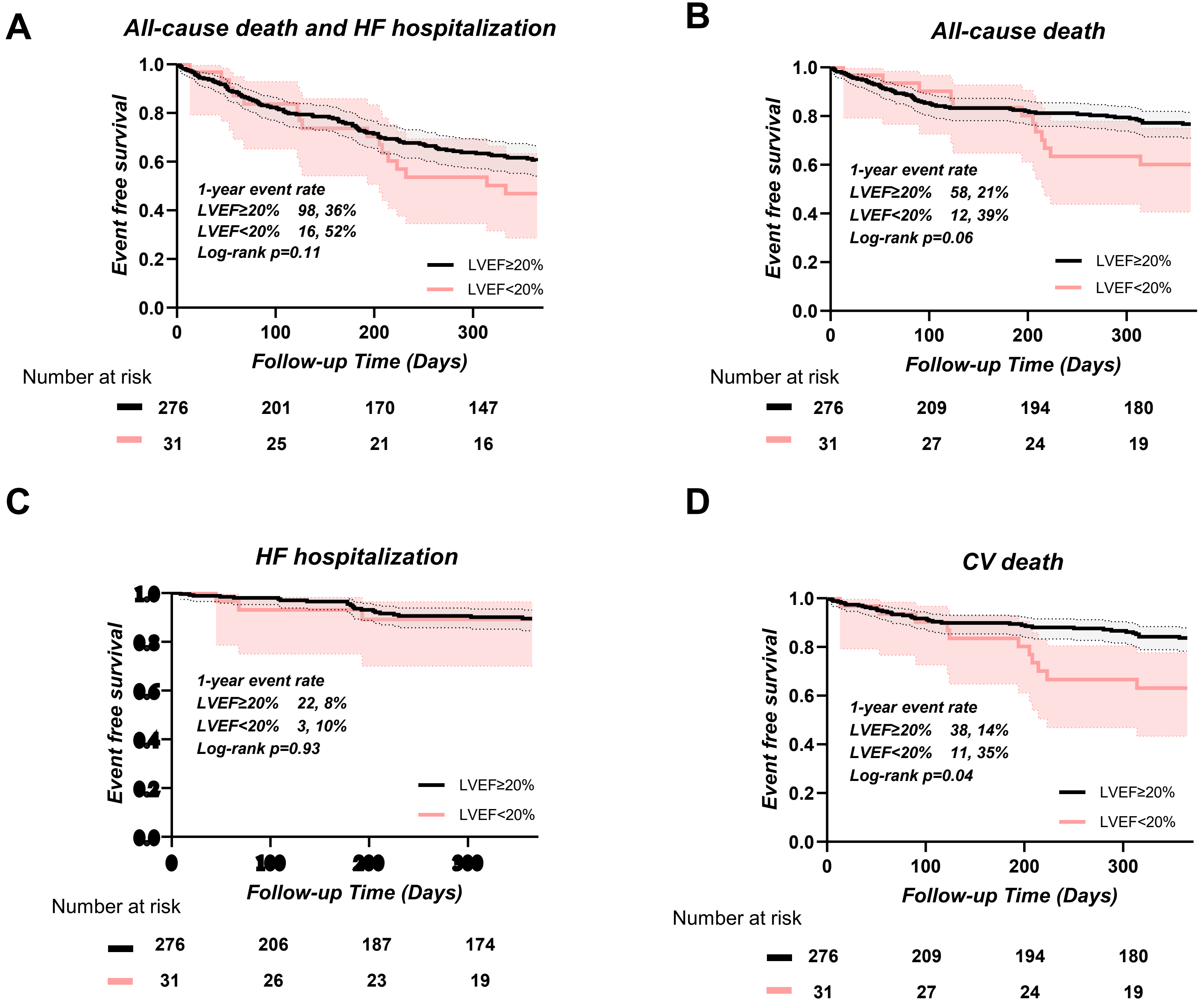

Supplemental Figure 4
